# 4517 Gender Disparities: Heart Failure in Puerto Rican Women

**DOI:** 10.1017/cts.2020.270

**Published:** 2020-07-29

**Authors:** Ariel Gonzalez-Cordero

**Affiliations:** 1 University of Puerto Rico-Medical Sciences Campus

## Abstract

OBJECTIVES/GOALS: Women within the ages of 65-75 have a lower incidence of heart failure than men.However,after the age of 75, the incidence of HF in womentriples,matchingthat of men.(Lloyd-Jones etal., 2002)Overall, women with heart failure live longer at the expense of presenting moresevere symptoms and poorer perceived quality of life.Generally, women with heart failurereceive suboptimal treatment throughout their lifetime. In fact, women are more likely todevelop heart failure after myocardial infarction. This trend is, in part, because physicians areless strict when treating them.(Chou et al., 2007)Studies in heart failure by ethnicity have shown that, despite equal access to healthcare, Hispanic women have higher rates of readmission than Non-Hispanic-white (NHW) women. (Durstenfeld, Ogedegbe, Katz, Park, &Blecker, 2016)One study in Boston demonstrated that Puerto Rican Women have higher rates of diabetes, obesity, and chronic kidney disease compared to blacks and NHW women.(Todorova, Tejada, & Castaneda-Sceppa, 2014)These are cardiovascular risk factors that warrant further study in Puerto Rican women living on the island, but data are lacking. Objective: The purpose of this study is to evaluate the gender disparities in presentation, management, and outcomes in Puerto Rican Hispanic hospitalized for heart failure METHODS/STUDY POPULATION: To this end, we will perform a secondary analysis of data from the PR CardiovascularSurveillance Study (PRCSS). We will extract personal data from 4,461 medical records of patients admitted with heart failure (ICD-9 Codes 428) at 21 hospitals in Puerto Rico, during theyears2007, 2009 and 2011. For statistical methods, we will implement chi-square and t-tests at a significance level of 0.05. RESULTS/ANTICIPATED RESULTS: We expect to find that women will have: fewer interventions, less optimized heart failure medication, higher BNP, older age of diagnosis, but paradoxically better outcome than male counterparts of the same age. DISCUSSION/SIGNIFICANCE OF IMPACT: With this study, we would like to raise awareness about gender-specific health disparities Puerto Rican Hispanic women with heart failure experience while hospitalized.

